# Dopaminium nitrate

**DOI:** 10.1107/S1600536814008265

**Published:** 2014-04-18

**Authors:** Sofian Gatfaoui, Houda Marouani, Thierry Roisnel, Hassouna Dhaouadi

**Affiliations:** a Laboratoire de Chimie des Matériaux, Faculté des Sciences de Bizerte, 7021 Zarzouna Bizerte, Tunisia; b Centre de Diffractométrie X, UMR 6226 CNRS, Unité Sciences Chimiques de Rennes, Université de Rennes I, 263 Avenue du Général Leclerc, 35042 Rennes, France; c Laboratoire des Matériaux Utiles, Institut National de Recherche et d’Analyse Physico-chimique, Pole Technologique de Sidi-Thabet, 2020 Tunis, Tunisia

## Abstract

The asymmetric unit of the title salt [systematic name: 2-(3,4-di­hydroxy­phen­yl)ethanaminium nitrate], C_8_H_12_NO_2_^+^·NO_3_^−^, contains two independent cations and two independent nitrate anions. The crystal structure consists of discrete nitrate ions stacked in layers parallel to (010). These layers are linked *via* the dopaminium cations by O—H⋯O, N—H⋯O and weak C—H⋯O hydrogen bonds, forming a three-dimensional supra­molecular network.

## Related literature   

For pharmacological properties of dopamine, see: Jones *et al.* (1999 ▶); Salamone & Correa (2002 ▶). For related structures, see: Gatfaoui *et al.* (2013 ▶, 2014*a* ▶, 2014*b* ▶); Marouani *et al.* (2012 ▶); Kefi *et al.* (2013 ▶). For the perchlorate salt of the title cation, see: Boghaei *et al.* (2008 ▶). For background to hydrogen bonding and aromatic π–π stacking inter­actions, see: Brown (1976 ▶); Blessing (1986 ▶); Janiak (2000 ▶).
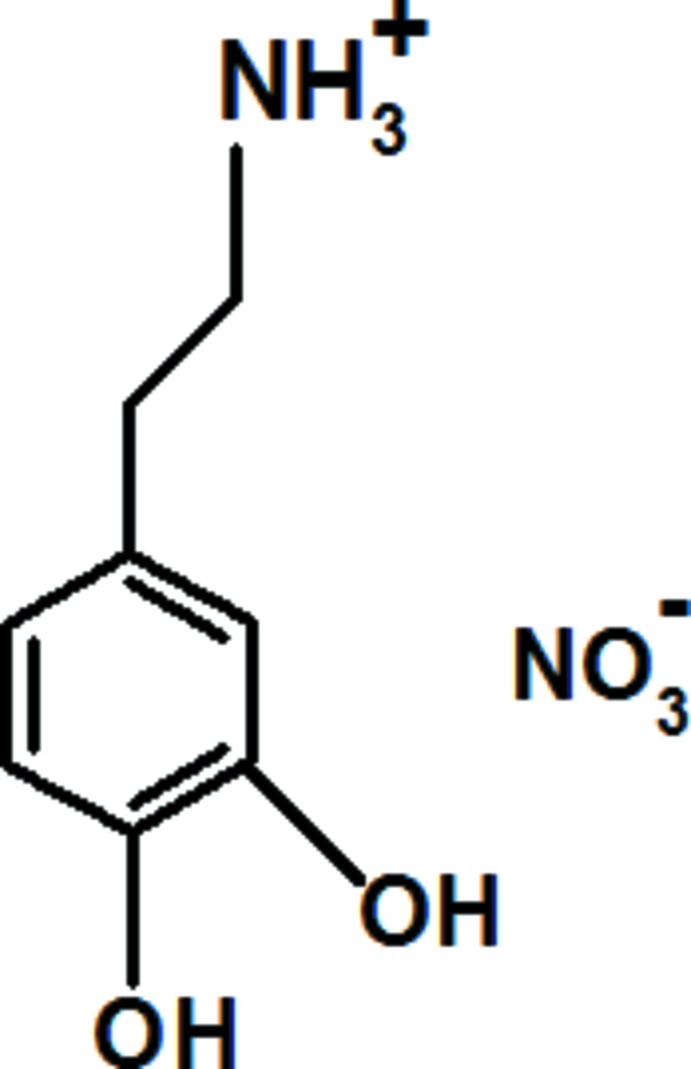


## Experimental   

### 

#### Crystal data  

C_8_H_12_NO_2_^+^·NO_3_^−^
*M* *_r_* = 216.20
Triclinic, 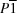

*a* = 8.3066 (4) Å
*b* = 10.4856 (5) Å
*c* = 11.2303 (7) Å
α = 79.623 (2)°
β = 89.868 (2)°
γ = 82.357 (2)°
*V* = 953.37 (9) Å^3^
*Z* = 4
Mo *K*α radiation
μ = 0.13 mm^−1^
*T* = 150 K
0.56 × 0.44 × 0.27 mm

#### Data collection  

Bruker APEXII diffractometer
Absorption correction: multi-scan (*SADABS*; Bruker, 2006 ▶) *T*_min_ = 0.870, *T*_max_ = 0.966
10787 measured reflections
4339 independent reflections
3583 reflections with *I* > 2σ(*I*)
*R*_int_ = 0.038

#### Refinement  

*R*[*F* ^2^ > 2σ(*F* ^2^)] = 0.043
*wR*(*F* ^2^) = 0.113
*S* = 1.05
4339 reflections
312 parameters
H atoms treated by a mixture of independent and constrained refinement
Δρ_max_ = 0.31 e Å^−3^
Δρ_min_ = −0.26 e Å^−3^

### 

Data collection: *APEX2* (Bruker, 2006 ▶); cell refinement: *SAINT* (Bruker, 2006 ▶); data reduction: *SAINT*; program(s) used to solve structure: *SIR97* (Altomare *et al.*, 1999 ▶); program(s) used to refine structure: *SHELXL97* (Sheldrick, 2008 ▶); molecular graphics: *ORTEP-3 for Windows* (Farrugia, 2012 ▶) and *DIAMOND* (Brandenburg & Putz, 2005 ▶); software used to prepare material for publication: *WinGX* (Farrugia, 2012 ▶) and *CRYSCAL* (T. Roisnel, local program).

## Supplementary Material

Crystal structure: contains datablock(s) I. DOI: 10.1107/S1600536814008265/bg2526sup1.cif

Structure factors: contains datablock(s) I. DOI: 10.1107/S1600536814008265/bg2526Isup2.hkl

Supporting information file. DOI: 10.1107/S1600536814008265/bg2526Isup3.cml

CCDC reference: 996988

Additional supporting information:  crystallographic information; 3D view; checkCIF report

## Figures and Tables

**Table 1:** Hydrogen-bond geometry (Å, °)

*D*—H⋯*A*	*D*—H	H⋯*A*	*D*⋯*A*	*D*—H⋯*A*
O1—H1*O*⋯O8^i^	0.86 (2)	1.96 (2)	2.7871 (15)	163 (2)
O2—H2*O*⋯O5^ii^	0.89 (2)	1.80 (2)	2.6863 (16)	169.7 (19)
O3—H3*O*⋯O7^iii^	0.89 (2)	1.94 (2)	2.8017 (15)	164 (2)
O4—H4*O*⋯O9^iv^	0.89 (2)	1.83 (2)	2.7196 (16)	176.6 (19)
N1—H1*N*⋯O1^iv^	0.94 (3)	2.10 (3)	3.0125 (19)	163.3 (19)
N1—H2*N*⋯O6^v^	0.90 (2)	2.10 (2)	2.9893 (17)	172.1 (17)
N1—H3*N*⋯O8^iv^	0.88 (2)	2.26 (2)	2.9867 (17)	139.6 (17)
N1—H3*N*⋯O2^vi^	0.88 (2)	2.42 (2)	3.0223 (16)	126.6 (15)
N2—H4*N*⋯O7^ii^	0.92 (2)	2.28 (2)	3.0020 (16)	135.2 (15)
N2—H4*N*⋯O8^i^	0.92 (2)	2.59 (2)	3.2672 (18)	131.3 (14)
N2—H5*N*⋯O10^vii^	0.93 (2)	1.98 (2)	2.9079 (17)	175.7 (18)
N2—H5*N*⋯O9^vii^	0.93 (2)	2.50 (2)	3.1400 (17)	126.3 (15)
N2—H6*N*⋯O6^viii^	0.93 (2)	2.17 (2)	2.8608 (17)	130.2 (15)
N2—H6*N*⋯O3^ii^	0.93 (2)	2.30 (2)	3.0236 (17)	134.5 (14)
C1—H1*B*⋯O5^v^	0.97	2.40	3.0783 (19)	126
C2—H2*A*⋯O4	0.97	2.46	3.4090 (19)	166

Symmetry codes: (i) 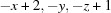; (ii) 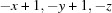; (iii) 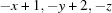; (iv) 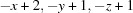; (v) 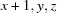; (vi) 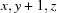; (vii) 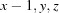; (viii) 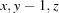.

**Fig. 1.**
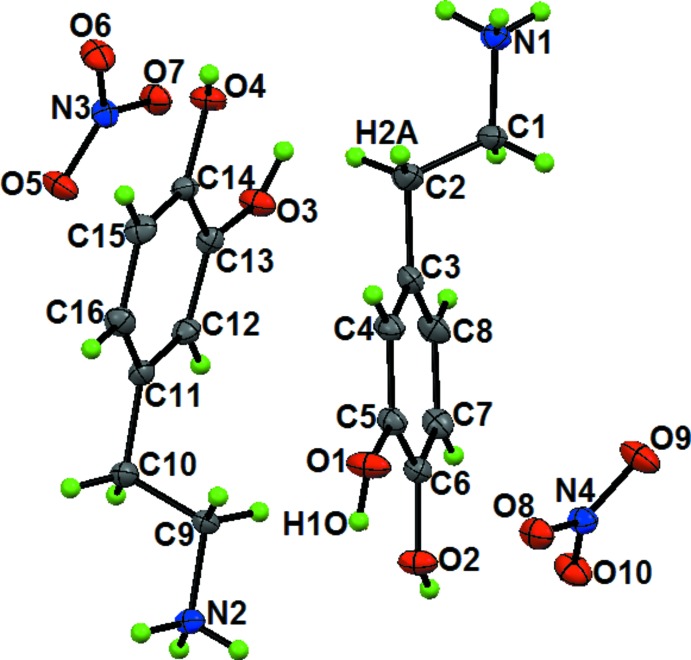
An ellipsoid plot of (I) with the atom-labelling scheme. Displacement ellipsoids are drawn at the 30% probability level.

**Fig. 2.**
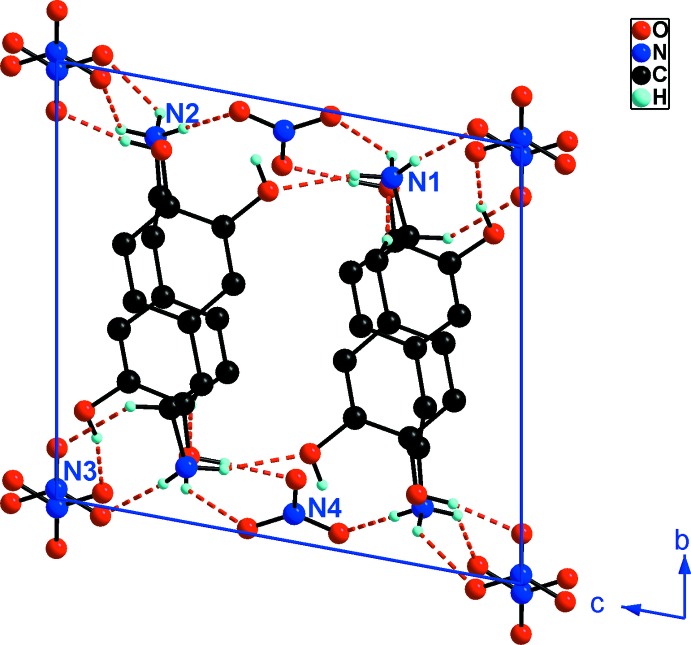
Projection of (I) along the a axis. The H-atoms not involved in H-bonding are omitted. N atoms labelled with their generic names (No symmetry codes applied)

## supplementary crystallographic information

### 1. Introduction

Dopamine is an important regulator of many physiological functions, including control of locomotion, cognition, affect, and neuroendocrine hormone secretion. The dopamine transporter (DAT) plays an important role in calibrating the duration and intensity of dopamine neurotransmission in the central nervous system (Jones *et al.*, 1999). In addition, dopamine is an important signal transmitter between the neurons and muscles (Salamone & Correa, 2002).

### 2.1. Synthesis and crystallization

An aqueous solution containing 1 mmol of HNO_3_ in 10 ml of water was added to 1 mmol of dopamine hydro­chloride in 10 ml of water. The obtained solution was stirred for 15 min and then left to stand at room temperature. Colorless single crystals of the title compound were obtained after some days.

### 2.2. Refinement

The hydrogen atoms bonded to oxygen and nitro­gen atoms were located from a difference map and were allowed to refine. The rest of the H atoms were treated as riding, with C—H = 0.97 Å (methyl­ene), or 0.93 Å (methine), with U_iso_(H) = 1.2U_eq_(C).

### 3. Results and discussion

In this work, we report the preparation and the structural investigation of the dopaminium nitrate, C_8_H_12_NO_2_·NO_3_ (I).

The asymmetric unit of (I) is composed of two independent dopaminium cations and two independent nitrate anions (Figure 1). The structure of the compound consists of discrete nitrate ions stacked in layers parallel to the (010) plane separated by organic cations (Figure 2). The structural cohesion is established by a three-dimensional network of N—H···O, O—H···O and weak C—H···O hydrogen bonds (Brown, 1976; Blessing, 1986).

Inter­atomic bond lengths and angles of the nitrate anions spread respectively within the ranges [1.2448 (16)–1.2596 (15) Å] and [119.50 (12)–120.87 (12)°]. These geometrical features have also been noticed in other crystal structures (Marouani *et al.*, 2012; Kefi *et al.*, 2013; Gatfaoui *et al.*, 2013, 2014*a*, 2014*b*).

In this atomic arrangement two independent dopaminium cations are present. Examination of the organic cations shows that the bond distances and angles show no significant difference from those obtained in other salt involving the same organic groups (Boghaei *et al.*, 2008). The aromatic rings are planar with an average deviation of 0.0014 Å and form a dihedral angle of 7.81°. The inter­planar distance between nearby phenyl rings is in the vicinity of 4.16 Å, a bit longer than required for a medium strength π–π inter­action (Janiak, 2000).

The established H-bonds of types O—H···O, N—H···O and C—H···O (Table 1) involve oxygen atoms of the nitrate anions as acceptors, and the protonated nitro­gen atoms, carbon and oxygen atoms of dopaminium as donors.

### Crystal data

**Table:**

C_8_H_12_NO_2_^+^·NO_3_^−^	*Z* = 4
*M**_r_* = 216.20	*F*(000) = 456
Triclinic, *P*1	*D*_x_ = 1.506 Mg m^−^^3^
Hall symbol: -P 1	Mo *K*α radiation, λ = 0.71073 Å
*a* = 8.3066 (4) Å	Cell parameters from 3958 reflections
*b* = 10.4856 (5) Å	θ = 2.5–27.5°
*c* = 11.2303 (7) Å	µ = 0.13 mm^−^^1^
α = 79.623 (2)°	*T* = 150 K
β = 89.868 (2)°	Prism, colorless
γ = 82.357 (2)°	0.56 × 0.44 × 0.27 mm
*V* = 953.37 (9) Å^3^	

### Data collection

**Table:**

Bruker APEXII diffractometer	3583 reflections with *I* > 2σ(*I*)
Graphite monochromator	*R*_int_ = 0.038
CCD rotation images, thin slices scans	θ_max_ = 27.5°, θ_min_ = 3.0°
Absorption correction: multi-scan (*SADABS*; Bruker, 2006)	*h* = −10→10
*T*_min_ = 0.870, *T*_max_ = 0.966	*k* = −13→13
10787 measured reflections	*l* = −14→14
4339 independent reflections	

### Refinement

**Table:**

Refinement on *F*^2^	Secondary atom site location: difference Fourier map
Least-squares matrix: full	Hydrogen site location: inferred from neighbouring sites
*R*[*F*^2^ > 2σ(*F*^2^)] = 0.043	H atoms treated by a mixture of independent and constrained refinement
*wR*(*F*^2^) = 0.113	*w* = 1/[σ^2^(*F*_o_^2^) + (0.0546*P*)^2^ + 0.2245*P*] where *P* = (*F*_o_^2^ + 2*F*_c_^2^)/3
*S* = 1.05	(Δ/σ)_max_ < 0.001
4339 reflections	Δρ_max_ = 0.31 e Å^−^^3^
312 parameters	Δρ_min_ = −0.26 e Å^−^^3^
0 restraints	Extinction correction: *SHELXL97* (Sheldrick, 2008), Fc^*^=kFc[1+0.001xFc^2^λ^3^/sin(2θ)]^-1/4^
Primary atom site location: structure-invariant direct methods	Extinction coefficient: 0.061 (4)

### Special details

**Table:**

Geometry. All e.s.d.'s (except the e.s.d. in the dihedral angle between two l.s. planes) are estimated using the full covariance matrix. The cell e.s.d.'s are taken into account individually in the estimation of e.s.d.'s in distances, angles and torsion angles; correlations between e.s.d.'s in cell parameters are only used when they are defined by crystal symmetry. An approximate (isotropic) treatment of cell e.s.d.'s is used for estimating e.s.d.'s involving l.s. planes.
Refinement. Refinement of *F*^2^ against ALL reflections. The weighted *R*-factor *wR* and goodness of fit *S* are based on *F*^2^, conventional *R*-factors *R* are based on *F*, with *F* set to zero for negative *F*^2^. The threshold expression of *F*^2^ > σ(*F*^2^) is used only for calculating *R*-factors(gt) *etc*. and is not relevant to the choice of reflections for refinement. *R*-factors based on *F*^2^ are statistically about twice as large as those based on *F*, and *R*- factors based on ALL data will be even larger.

### Fractional atomic coordinates and isotropic or equivalent isotropic displacement parameters (Å^2^)

**Table:**

	*x*	*y*	*z*	*U*_iso_*/*U*_eq_	
O1	0.88256 (15)	0.20879 (11)	0.45485 (10)	0.0270 (3)	
O2	0.89365 (13)	0.15989 (10)	0.22563 (10)	0.0231 (3)	
O3	0.52188 (13)	0.78884 (10)	0.05973 (9)	0.0218 (2)	
O4	0.50913 (13)	0.84937 (9)	0.28522 (10)	0.0199 (2)	
O5	0.13563 (13)	0.88517 (10)	0.00083 (10)	0.0240 (3)	
O6	0.23349 (13)	1.02198 (11)	0.09420 (10)	0.0247 (3)	
O7	0.27175 (12)	1.03129 (10)	−0.09879 (9)	0.0212 (2)	
O8	1.24531 (13)	0.01122 (10)	0.58958 (10)	0.0240 (3)	
O9	1.39421 (14)	0.14414 (11)	0.48499 (10)	0.0283 (3)	
O10	1.24180 (13)	0.03858 (11)	0.39320 (10)	0.0264 (3)	
N1	1.03491 (17)	0.87415 (12)	0.27899 (14)	0.0202 (3)	
N2	0.47018 (16)	0.13341 (12)	0.21268 (13)	0.0181 (3)	
N3	0.21404 (14)	0.97956 (11)	−0.00085 (11)	0.0170 (3)	
N4	1.29264 (14)	0.06439 (11)	0.48912 (11)	0.0189 (3)	
C1	1.02008 (18)	0.74577 (14)	0.24467 (15)	0.0226 (3)	
H1A	1.1233	0.6897	0.2606	0.027*	
H1B	0.9964	0.7590	0.1584	0.027*	
C2	0.89005 (19)	0.67827 (14)	0.31175 (16)	0.0252 (4)	
H2A	0.7850	0.7292	0.2887	0.030*	
H2B	0.9067	0.6732	0.3980	0.030*	
C3	0.89030 (17)	0.54144 (14)	0.28525 (14)	0.0204 (3)	
C4	0.88790 (18)	0.43580 (14)	0.38027 (14)	0.0208 (3)	
H4	0.8855	0.4503	0.4596	0.025*	
C5	0.88905 (17)	0.30931 (14)	0.35856 (13)	0.0187 (3)	
C6	0.89502 (16)	0.28653 (13)	0.23989 (14)	0.0175 (3)	
C7	0.89493 (18)	0.39103 (14)	0.14486 (14)	0.0211 (3)	
H7	0.8966	0.3767	0.0655	0.025*	
C8	0.89233 (18)	0.51738 (15)	0.16758 (15)	0.0226 (3)	
H8	0.8919	0.5868	0.1030	0.027*	
C9	0.49926 (16)	0.26836 (13)	0.22214 (13)	0.0163 (3)	
H9A	0.5741	0.2984	0.1602	0.020*	
H9B	0.5490	0.2672	0.3005	0.020*	
C10	0.34207 (17)	0.36269 (13)	0.20725 (14)	0.0191 (3)	
H10A	0.2969	0.3709	0.1262	0.023*	
H10B	0.2634	0.3298	0.2648	0.023*	
C11	0.37719 (16)	0.49492 (13)	0.22873 (14)	0.0178 (3)	
C12	0.42284 (16)	0.58718 (13)	0.13394 (13)	0.0177 (3)	
H12	0.4214	0.5706	0.0553	0.021*	
C13	0.47037 (16)	0.70326 (13)	0.15462 (13)	0.0160 (3)	
C14	0.46516 (16)	0.73132 (13)	0.27195 (13)	0.0159 (3)	
C15	0.41976 (17)	0.64019 (14)	0.36697 (13)	0.0199 (3)	
H15	0.4173	0.6580	0.4452	0.024*	
C16	0.37771 (18)	0.52191 (14)	0.34542 (14)	0.0207 (3)	
H16	0.3497	0.4604	0.4098	0.025*	
H1N	1.070 (3)	0.864 (2)	0.360 (2)	0.053 (7)*	
H2N	1.098 (2)	0.9221 (19)	0.2291 (18)	0.033 (5)*	
H3N	0.941 (2)	0.9239 (19)	0.2786 (17)	0.034 (5)*	
H4N	0.565 (2)	0.0765 (17)	0.2227 (16)	0.028 (5)*	
H5N	0.402 (2)	0.1015 (19)	0.2728 (19)	0.035 (5)*	
H6N	0.424 (2)	0.1300 (17)	0.1380 (18)	0.028 (5)*	
H1O	0.857 (3)	0.142 (2)	0.429 (2)	0.053 (7)*	
H2O	0.887 (2)	0.154 (2)	0.147 (2)	0.043 (6)*	
H3O	0.578 (3)	0.845 (2)	0.0863 (19)	0.047 (6)*	
H4O	0.538 (2)	0.8496 (19)	0.361 (2)	0.040 (6)*	

### Atomic displacement parameters (Å^2^)

**Table:**

	*U*^11^	*U*^22^	*U*^33^	*U*^12^	*U*^13^	*U*^23^
O1	0.0489 (7)	0.0187 (6)	0.0161 (6)	−0.0126 (5)	0.0031 (5)	−0.0042 (4)
O2	0.0371 (6)	0.0168 (5)	0.0170 (6)	−0.0066 (4)	0.0011 (5)	−0.0051 (4)
O3	0.0335 (6)	0.0192 (5)	0.0148 (5)	−0.0108 (4)	0.0025 (5)	−0.0032 (4)
O4	0.0293 (6)	0.0146 (5)	0.0168 (6)	−0.0056 (4)	−0.0013 (4)	−0.0040 (4)
O5	0.0315 (6)	0.0245 (6)	0.0198 (6)	−0.0165 (5)	0.0031 (5)	−0.0054 (4)
O6	0.0280 (6)	0.0328 (6)	0.0181 (6)	−0.0118 (5)	0.0017 (5)	−0.0117 (5)
O7	0.0263 (5)	0.0209 (5)	0.0172 (6)	−0.0081 (4)	0.0057 (4)	−0.0018 (4)
O8	0.0325 (6)	0.0241 (6)	0.0175 (6)	−0.0111 (4)	0.0084 (5)	−0.0043 (4)
O9	0.0385 (6)	0.0309 (6)	0.0200 (6)	−0.0218 (5)	0.0027 (5)	−0.0046 (5)
O10	0.0323 (6)	0.0329 (6)	0.0181 (6)	−0.0137 (5)	0.0002 (5)	−0.0082 (5)
N1	0.0240 (7)	0.0153 (6)	0.0222 (7)	−0.0058 (5)	0.0039 (6)	−0.0034 (5)
N2	0.0231 (6)	0.0141 (6)	0.0176 (7)	−0.0031 (5)	0.0019 (5)	−0.0039 (5)
N3	0.0166 (5)	0.0187 (6)	0.0159 (6)	−0.0028 (4)	−0.0006 (5)	−0.0031 (5)
N4	0.0230 (6)	0.0173 (6)	0.0172 (7)	−0.0051 (5)	0.0033 (5)	−0.0037 (5)
C1	0.0250 (7)	0.0161 (7)	0.0296 (9)	−0.0070 (6)	0.0078 (6)	−0.0089 (6)
C2	0.0281 (8)	0.0179 (7)	0.0316 (9)	−0.0076 (6)	0.0107 (7)	−0.0071 (6)
C3	0.0178 (7)	0.0189 (7)	0.0265 (8)	−0.0072 (5)	0.0052 (6)	−0.0059 (6)
C4	0.0251 (7)	0.0211 (7)	0.0195 (8)	−0.0093 (6)	0.0063 (6)	−0.0080 (6)
C5	0.0214 (7)	0.0184 (7)	0.0172 (7)	−0.0066 (5)	0.0030 (6)	−0.0030 (6)
C6	0.0172 (6)	0.0169 (7)	0.0198 (8)	−0.0046 (5)	0.0007 (6)	−0.0053 (6)
C7	0.0233 (7)	0.0250 (8)	0.0167 (7)	−0.0080 (6)	0.0013 (6)	−0.0045 (6)
C8	0.0259 (8)	0.0185 (7)	0.0233 (8)	−0.0083 (6)	0.0022 (6)	0.0004 (6)
C9	0.0167 (6)	0.0133 (6)	0.0195 (7)	−0.0044 (5)	0.0008 (6)	−0.0030 (5)
C10	0.0179 (7)	0.0149 (7)	0.0251 (8)	−0.0027 (5)	−0.0003 (6)	−0.0044 (6)
C11	0.0152 (6)	0.0133 (6)	0.0247 (8)	−0.0006 (5)	−0.0001 (6)	−0.0036 (6)
C12	0.0191 (7)	0.0181 (7)	0.0169 (7)	−0.0014 (5)	0.0004 (6)	−0.0066 (6)
C13	0.0169 (6)	0.0145 (6)	0.0150 (7)	−0.0005 (5)	−0.0002 (5)	−0.0002 (5)
C14	0.0171 (6)	0.0125 (6)	0.0184 (7)	−0.0006 (5)	−0.0011 (5)	−0.0044 (5)
C15	0.0259 (7)	0.0199 (7)	0.0138 (7)	−0.0030 (6)	0.0021 (6)	−0.0033 (6)
C16	0.0242 (7)	0.0164 (7)	0.0203 (8)	−0.0033 (6)	0.0029 (6)	0.0005 (6)

### Geometric parameters (Å, º)

**Table:**

O1—C5	1.3738 (18)	C2—H2A	0.9700
O1—H1O	0.86 (2)	C2—H2B	0.9700
O2—C6	1.3674 (16)	C3—C8	1.389 (2)
O2—H2O	0.89 (2)	C3—C4	1.395 (2)
O3—C13	1.3709 (17)	C4—C5	1.3899 (19)
O3—H3O	0.89 (2)	C4—H4	0.9300
O4—C14	1.3693 (16)	C5—C6	1.395 (2)
O4—H4O	0.89 (2)	C6—C7	1.385 (2)
O5—N3	1.2526 (15)	C7—C8	1.391 (2)
O6—N3	1.2467 (15)	C7—H7	0.9300
O7—N3	1.2592 (15)	C8—H8	0.9300
O8—N4	1.2522 (15)	C9—C10	1.5196 (18)
O9—N4	1.2596 (15)	C9—H9A	0.9700
O10—N4	1.2448 (16)	C9—H9B	0.9700
N1—C1	1.4856 (18)	C10—C11	1.5147 (18)
N1—H1N	0.94 (3)	C10—H10A	0.9700
N1—H2N	0.90 (2)	C10—H10B	0.9700
N1—H3N	0.88 (2)	C11—C16	1.390 (2)
N2—C9	1.4889 (17)	C11—C12	1.392 (2)
N2—H4N	0.915 (19)	C12—C13	1.3862 (19)
N2—H5N	0.93 (2)	C12—H12	0.9300
N2—H6N	0.93 (2)	C13—C14	1.400 (2)
C1—C2	1.497 (2)	C14—C15	1.386 (2)
C1—H1A	0.9700	C15—C16	1.394 (2)
C1—H1B	0.9700	C15—H15	0.9300
C2—C3	1.5166 (19)	C16—H16	0.9300
			
C5—O1—H1O	109.1 (15)	O1—C5—C6	121.11 (13)
C6—O2—H2O	111.0 (13)	C4—C5—C6	119.73 (13)
C13—O3—H3O	110.9 (14)	O2—C6—C7	124.11 (13)
C14—O4—H4O	111.6 (13)	O2—C6—C5	116.37 (13)
C1—N1—H1N	111.3 (13)	C7—C6—C5	119.46 (13)
C1—N1—H2N	113.2 (12)	C6—C7—C8	120.29 (14)
H1N—N1—H2N	110.9 (18)	C6—C7—H7	119.9
C1—N1—H3N	113.0 (12)	C8—C7—H7	119.9
H1N—N1—H3N	102.2 (18)	C3—C8—C7	121.00 (14)
H2N—N1—H3N	105.6 (17)	C3—C8—H8	119.5
C9—N2—H4N	111.3 (11)	C7—C8—H8	119.5
C9—N2—H5N	110.8 (12)	N2—C9—C10	111.48 (11)
H4N—N2—H5N	106.3 (16)	N2—C9—H9A	109.3
C9—N2—H6N	112.1 (11)	C10—C9—H9A	109.3
H4N—N2—H6N	107.8 (16)	N2—C9—H9B	109.3
H5N—N2—H6N	108.3 (16)	C10—C9—H9B	109.3
O6—N3—O5	119.78 (12)	H9A—C9—H9B	108.0
O6—N3—O7	120.57 (11)	C11—C10—C9	108.97 (11)
O5—N3—O7	119.65 (12)	C11—C10—H10A	109.9
O10—N4—O8	120.87 (12)	C9—C10—H10A	109.9
O10—N4—O9	119.50 (12)	C11—C10—H10B	109.9
O8—N4—O9	119.63 (12)	C9—C10—H10B	109.9
N1—C1—C2	112.95 (12)	H10A—C10—H10B	108.3
N1—C1—H1A	109.0	C16—C11—C12	118.72 (13)
C2—C1—H1A	109.0	C16—C11—C10	120.30 (13)
N1—C1—H1B	109.0	C12—C11—C10	120.75 (13)
C2—C1—H1B	109.0	C13—C12—C11	121.16 (13)
H1A—C1—H1B	107.8	C13—C12—H12	119.4
C1—C2—C3	111.68 (12)	C11—C12—H12	119.4
C1—C2—H2A	109.3	O3—C13—C12	119.41 (13)
C3—C2—H2A	109.3	O3—C13—C14	121.02 (12)
C1—C2—H2B	109.3	C12—C13—C14	119.57 (13)
C3—C2—H2B	109.3	O4—C14—C15	123.85 (13)
H2A—C2—H2B	107.9	O4—C14—C13	116.47 (12)
C8—C3—C4	118.26 (13)	C15—C14—C13	119.67 (13)
C8—C3—C2	121.71 (14)	C14—C15—C16	120.06 (14)
C4—C3—C2	120.03 (14)	C14—C15—H15	120.0
C5—C4—C3	121.22 (14)	C16—C15—H15	120.0
C5—C4—H4	119.4	C11—C16—C15	120.73 (13)
C3—C4—H4	119.4	C11—C16—H16	119.6
O1—C5—C4	119.15 (13)	C15—C16—H16	119.6
			
N1—C1—C2—C3	−173.68 (13)	N2—C9—C10—C11	175.05 (12)
C1—C2—C3—C8	−49.6 (2)	C9—C10—C11—C16	−86.05 (16)
C1—C2—C3—C4	130.67 (15)	C9—C10—C11—C12	88.48 (16)
C8—C3—C4—C5	0.6 (2)	C16—C11—C12—C13	0.9 (2)
C2—C3—C4—C5	−179.61 (13)	C10—C11—C12—C13	−173.70 (12)
C3—C4—C5—O1	−178.42 (13)	C11—C12—C13—O3	176.89 (12)
C3—C4—C5—C6	0.9 (2)	C11—C12—C13—C14	−3.1 (2)
O1—C5—C6—O2	0.1 (2)	O3—C13—C14—O4	1.94 (19)
C4—C5—C6—O2	−179.17 (13)	C12—C13—C14—O4	−178.10 (12)
O1—C5—C6—C7	177.44 (13)	O3—C13—C14—C15	−177.00 (13)
C4—C5—C6—C7	−1.9 (2)	C12—C13—C14—C15	3.0 (2)
O2—C6—C7—C8	178.41 (13)	O4—C14—C15—C16	−179.61 (13)
C5—C6—C7—C8	1.3 (2)	C13—C14—C15—C16	−0.7 (2)
C4—C3—C8—C7	−1.2 (2)	C12—C11—C16—C15	1.3 (2)
C2—C3—C8—C7	179.05 (13)	C10—C11—C16—C15	175.98 (13)
C6—C7—C8—C3	0.2 (2)	C14—C15—C16—C11	−1.4 (2)

### Hydrogen-bond geometry (Å, º)

**Table:**

*D*—H···*A*	*D*—H	H···*A*	*D*···*A*	*D*—H···*A*
O1—H1*O*···O8^i^	0.86 (2)	1.96 (2)	2.7871 (15)	163 (2)
O2—H2*O*···O5^ii^	0.89 (2)	1.80 (2)	2.6863 (16)	169.7 (19)
O3—H3*O*···O7^iii^	0.89 (2)	1.94 (2)	2.8017 (15)	164 (2)
O4—H4*O*···O9^iv^	0.89 (2)	1.83 (2)	2.7196 (16)	176.6 (19)
N1—H1*N*···O1^iv^	0.94 (3)	2.10 (3)	3.0125 (19)	163.3 (19)
N1—H2*N*···O6^v^	0.90 (2)	2.10 (2)	2.9893 (17)	172.1 (17)
N1—H3*N*···O8^iv^	0.88 (2)	2.26 (2)	2.9867 (17)	139.6 (17)
N1—H3*N*···O2^vi^	0.88 (2)	2.42 (2)	3.0223 (16)	126.6 (15)
N2—H4*N*···O7^ii^	0.92 (2)	2.28 (2)	3.0020 (16)	135.2 (15)
N2—H4*N*···O8^i^	0.92 (2)	2.59 (2)	3.2672 (18)	131.3 (14)
N2—H5*N*···O10^vii^	0.93 (2)	1.98 (2)	2.9079 (17)	175.7 (18)
N2—H5*N*···O9^vii^	0.93 (2)	2.50 (2)	3.1400 (17)	126.3 (15)
N2—H6*N*···O6^viii^	0.93 (2)	2.17 (2)	2.8608 (17)	130.2 (15)
N2—H6*N*···O3^ii^	0.93 (2)	2.30 (2)	3.0236 (17)	134.5 (14)
C1—H1*B*···O5^v^	0.97	2.40	3.0783 (19)	126
C2—H2*A*···O4	0.97	2.46	3.4090 (19)	166

Symmetry codes: (i) −*x*+2, −*y*, −*z*+1; (ii) −*x*+1, −*y*+1, −*z*; (iii) −*x*+1, −*y*+2, −*z*; (iv) −*x*+2, −*y*+1, −*z*+1; (v) *x*+1, *y*, *z*; (vi) *x*, *y*+1, *z*; (vii) *x*−1, *y*, *z*; (viii) *x*, *y*−1, *z*.
